# Psychosocial Problems, Indoor Air-Related Symptoms, and Perceived Indoor Air Quality among Students in Schools without Indoor Air Problems: A Longitudinal Study

**DOI:** 10.3390/ijerph15071497

**Published:** 2018-07-16

**Authors:** Eerika Finell, Asko Tolvanen, Juha Pekkanen, Jaana Minkkinen, Timo Ståhl, Arja Rimpelä

**Affiliations:** 1 Faculty of Social Sciences (Social Sciences), University of Tampere, 33014 Tampere, Finland; 2 Methodology Centre for Human Sciences, University of Jyväskylä, 40014 Jyväskylä, Finland; asko.j.tolvanen@jyu.fi; 3 Department of Public Health, University of Helsinki, 00014 Helsinki, Finland; juha.pekkanen@helsinki.fi; 4 Department of Health Security, National Institute for Health and Welfare, 70701 Kuopio, Finland; 5 Faculty of Social Sciences (Psychology), University of Tampere, 33014 Tampere, Finland; jaana.minkkinen@uta.fi; 6 Department of Welfare, National Institute for Health and Welfare, 33520 Tampere, Finland; Timo.Stahl@thl.fi; 7 Faculty of Social Sciences (Health Sciences), University of Tampere, 33014 Tampere, Finland; arja.rimpela@uta.fi; 8 PERLA (Tampere Centre for Childhood, Youth and Family Research), University of Tampere, 33014 Tampere, Finland; 9 Department of Adolescent Psychiatry, Tampere University Hospital, 33380 Nokia, Finland

**Keywords:** indoor air problems, indoor air quality, psychosocial problems, socioemotional difficulties, indoor air-related symptoms, teacher–student relations, lower secondary schools

## Abstract

The effect of students’ psychosocial problems on their reporting of indoor air quality (subjective IAQ) and indoor air-related (IA-related) symptoms has not been studied in schools in a longitudinal setting. Therefore, we analyzed whether changes in students’ psychosocial problems (socioemotional difficulties and perceived teacher–student relations) between the beginning of seventh grade (age 12–13 years) and the end of ninth grade (15–16 years) predicted subjective IAQ and IA-related symptoms at the end of ninth grade. In order to explore the independent effect of psychosocial factors, we focused only on students in schools without observed indoor air problems. The analysis was of longitudinal data (*N* = 986 students) using latent change modelling. Increased socioemotional difficulties were related to more IA-related symptoms (standardized beta = 0.20) and deteriorated subjective IAQ (standardized beta = 0.20). Increased problems in teacher–student relations were related to deteriorated subjective IAQ (standardized beta = 0.21). Although students’ psychosocial problems explained only 9–13% of the total variances, our findings support the notion that psychosocial factors also need to be taken into account in the evaluation of IAQ and the prevalence of IA-related symptoms in schools.

## 1. Introduction

Questionnaires mapping the subjective evaluation of indoor air quality (subjective IAQ) and the prevalence of indoor air-related (IA-related) symptoms such as reported respiratory symptoms are often used to evaluate the quality of indoor environments [1,2,3,4]. Therefore, an important question is: how well do self-reported IAQ and IA-related symptoms reflect problems in the quality of indoor air, and to what degree do they reflect other factors such as psychosocial problems?

There are well-documented relationships between building-related factors and subjective IAQ [5,6,7] as well as between building-related factors and the prevalence of IA-related symptoms [8,9,10]. Furthermore, psychosocial problems seem to be related to subjective IAQ and IA-related symptoms. For example, poor work support, high work demands, high stress and perceived control over indoor climate are associated with decreased subjective IAQ in workplaces [11,12,13]. There is also evidence that psychological processes and disorders are associated with IA-related symptoms such as symptoms of rhinitis [14,15,16,17] and asthma [18,19,20,21]. A limitation of previous research, however, is that it focuses mainly on adult populations; our knowledge about other age groups such as school-age children is inadequate.

### 1.1. Psychosocial Problems, Subjective IAQ, and IA-Related Symptoms in Schools

Problem in schools’ indoor environments can have many adverse consequences for students, such as negative health effects [22,23], lower academic performance [24], increased absenteeism [25], and poor student-perceived social climate [26].

Despite the fact that problems in schools’ indoor environments are relatively common in many countries [22,27,28], most of the research on the association between psychosocial problems, subjective IAQ and IA-related symptoms in schools has been conducted among teachers [29,30,31]. Only one cross-sectional study to date has analyzed whether and how psychosocial problems are related to subjective IAQ among students [32]. Furthermore, only one cross-sectional study has analyzed the relationship between students’ psychosocial problems and their IA-related symptoms [33]. Given that adolescents’ psychosocial problems often reflect perceived stress and strain [34,35,36], it is likely that these problems are associated with these factors. School stress has already been shown to be associated with deteriorated subjective IAQ among students [32]. Furthermore, among adults and children alike, psychological stress is associated with symptoms of rhinitis and asthma [18,37,38].

### 1.2. Present Study

The aims of the present study are to analyze the above issues among lower secondary school students living in the metropolitan area of Helsinki, Finland. The psychosocial problems on which we focus are students’ self-reported socioemotional difficulties and perceptions of teacher–student relations. Previous findings on the association between psychosocial factors and subjective IAQ have been based on cross-sectional data among both adults and children [11,32]. Instead, we analyze whether the change in students’ socioemotional difficulties and perceived teacher–student relations between seventh grade (age 12–13 years) and ninth grade (15–16 years) is related to their reported IA-related symptoms and subjective IAQ in ninth grade.

Given that indoor air (IA) problems can also create psychosocial problems—for example, by evoking inter-individual conflicts [39,40] or issues arising from building-related health problems [41]—the relationship between psychosocial problems and subjective IAQ might be bidirectional. Furthermore, there is some evidence that the relationship between psychosocial problems and IA-related symptoms is also bidirectional [19,20,42]. It follows that we cannot analyze the independent effect of psychosocial problems on our outcome variables in the presence of IA problems. Therefore, we focus only on students in schools where there are no such problems. This allows us to better understand how psychosocial problems are associated with our outcome variables, in a context where these associations are not affected by a third factor, namely health or social problems induced by a problematic indoor environment. This design has also been used in previous indoor environmental research [11].

## 2. Materials and Methods

### 2.1. Participants and Data Collection

Classroom online survey data were collected in two waves from all lower secondary school students at the beginning of seventh (2011; i.e., Time 1) and the end of ninth grade (2014; i.e., Time 2) in the metropolitan area of Helsinki (MetLoFIN). At baseline the students were 12–13 years old (*N* = 9497, response rate 73% of original cohort); at follow-up they were 15–16 years old (*N* = 5742, response rate 60.5% of baseline survey). This study analyzes only the responses of those who completed the surveys in both years (see Kinnunen et al. [43]; Minkkinen et al. [44]).

### 2.2. Ethical Considerations

The study protocol was approved by the Ethical Committee of the National Institute of Health and Welfare in 2011 (statement code 27.5.2011) and 2014 (9.4.2014). Educational authorities in 14 municipalities of the metropolitan area gave permission for the study. Parental consent was not required, as the data were gathered as part of school routine. However, two of the municipalities made parental consent statements obligatory, and these were collected. An information letter was sent to parents in the remaining 12 municipalities. Students’ participation was voluntary, and this was stated in the questionnaire instructions.

### 2.3. Measures

#### 2.3.1. Outcome Variables (Time 2)

The dependent variables were the subjective evaluation of IAQ (subjective IAQ) and IA-related symptoms. Subjective IAQ was measured by three items: “In your school, have the following conditions disturbed your schoolwork during last month? (a) Stuffy (bad) air; (b) dust, dirt; (c) unpleasant odour.” These items were measured on a four-point scale (1 = not at all, 2 = a little, 3 = quite a lot, 4 = very much). A mean score for the items were calculated. If the respondent answered fewer than two items, the score was not calculated. The reliability was good (Cronbach’s alpha = 0.88). Similar indices have also been used in previous studies [45]. The second outcome variable, IA-related symptoms, was measured by five items: “Have you had the following symptoms during the last six months? (a) Stuffy nose or rhinitis, (b) raspy voice, (c) cough, (d) dyspnea, (e) itchy or watering eyes.” These items were measured on a four-point scale (1 = seldom or never, 2 = about once month, 3 = about once a week, 4 = almost every day). If the respondent answered at least three items, a mean score for those items were calculated. The reliability of IA-related symptoms was good (Cronbach’s alpha = 0.81).

#### 2.3.2. Predictors (Times 1 and 2)

We used the Strength and Difficulties Questionnaire (SDQ) to measure the students’ socioemotional difficulties [46]. The SDQ is a brief instrument for screening emotional and behavioral problems in children and adolescents; the reliability and validity of this instrument have been shown to be high, including in Finland [47,48]. The SDQ [46] includes 25 items divided into five subscales covering behavioral problems, emotional symptoms, hyperactivity and inattention, peer relationship problems, and prosocial behaviors. For each item the options include a three-point scale (0 = not true, 1 = somewhat true, 2 = entirely true). The item ratings for four of the subscales (excluding prosocial behaviors) were added together to achieve a total difficulties score ranging from 0 to 40, following the instructions provided by Goodman, Meltzer, and Bailey [46]. Cronbach’s alpha was 0.67 for Time 1 and 0.73 for Time 2. We measured the perceived quality of teacher–student relations by four items: “Teachers encourage me to express my opinion in the classroom.”; “Teachers are interested in how I am doing.”; “Teachers treat us students fairly.”; and “The opinions of students are taken into consideration in the development of schoolwork.” The response scale ranged from 1 (strongly agree) to 5 (strongly disagree). The mean score was calculated in the same way as for IA-related symptoms. The reliability of this variable was good (Time 1: Cronbach’s alpha = 0.79; Time 2: Cronbach’s alpha = 0.83). These items have been used in many previous studies as indicators of teacher–student relations [26,49,50].

#### 2.3.3. Background Variables (Times 1 and 2)

The background variables included gender (0 = female, 1 = male), ethnic background (0 = speaks Finnish or Swedish with family members, 1 = speaks other languages), parents’ highest education (0 = low, 1 = middle, 2 = high education) and asthma/allergy (i.e., whether the student suffered from asthma or allergies or both). We used two items to construct this variable. For Time 1 we used the following two items: “Do you have any long-term illness or disability? (a) Asthma, (b) allergic rhinitis or other allergy.” The latter item was replaced in 2014 with the item “allergic rhinitis or hay fever”. The items were recoded so that a value of 0 indicated no asthma/allergy and a value of 1 indicated asthma and/or allergy.

### 2.4. Defining Schools without IA Problems

As explained in the introduction, we strove to focus only on schools without IA problems. Official inspections of health and safety in the school environment and the well-being of the school community are required under Healthcare Act 1326/2010, which states that all schools in Finland must be checked in every three years. This is done in cooperation with a number of authorities, such as representatives from the health authority and authorities responsible for the construction and maintenance of school buildings. The inspection of the indoor environment is large scale and includes a review of existing building-related documents such as results from any IAQ measurements and questionnaires, and a building walk-through focusing on areas where problems have been found or suspected [32,51].

To identify schools without IA problems, we used Benchmarking System of Health Promotion Capacity-Building (BSHPCB) data sets from comprehensive schools in 2011, 2013, and 2015. From the BSHPCB 2011 and 2013 data, we used a variable that measured whether any “biological exposures (e.g., indoor air, mold)” had been observed in the school during the most recent inspection. The response options were: 1 = no data available; 2 = not included in the inspection; 3 = inspected, no deficiencies detected; 4 = inspected, deficiencies detected but not yet corrected; 5 = inspected, deficiencies detected and corrected. This variable has also been used as an indicator of IA problems in previous research [26,32]. From the BSHPCB 2015 data, we used a variable that measured whether mold or damp had been observed in the school during the most recent inspection. The response options were the same as in 2011 and 2013. Only schools that reported that the inspection had been done and no deficiencies detected in all three years were selected. One year without information was allowed.

Only 20 out of 123 schools met our criteria (*N* = 1061 students). Students that had changed school during their lower secondary years were excluded from the analysis (*N* = 74). In addition, we excluded one univariate outlier (socioemotional difficulties *z*-score > 4). The final sample was 986 students.

### 2.5. Statistical Analysis

In order to answer our research questions, we built and then analyzed eight linear regression models and two latent change models [52]. As an estimator, we used a full information maximum likelihood estimation (FIML) with robust standard errors (MLR in Mplus). We computed MLR standard errors using a sandwich estimator that was robust to violations of non-normality and non-independence of observation (i.e., complex model in Mplus) [53]. Our observations were non-independent because our data were hierarchical (schoolchildren nested within schools). We could not use a multilevel approach except in null models because we had only 20 schools at cluster level. Such a small sample size can produce biased standard errors at cluster level if a multilevel approach is used [54]. Mplus statistical software 7.0 (Muthen & Muthen, Los Angeles, CA, USA) was used in the analysis.

**Figure 1:**
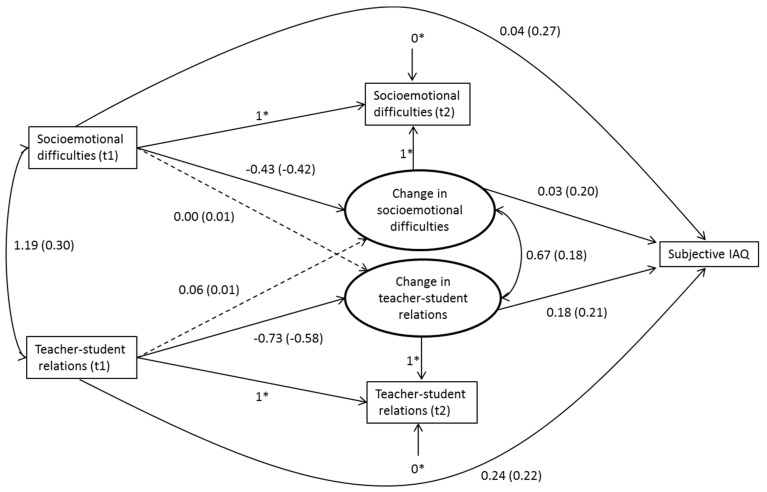
Unstandardized regression coefficients of latent change factor model: subjective IAQ on socioemotional difficulties and perceived teacher–student relations (*N* = 986). Standardized regression coefficients in parentheses. Dashed lines represent non-significant associations. * Fixed factors.

**Figure 2:**
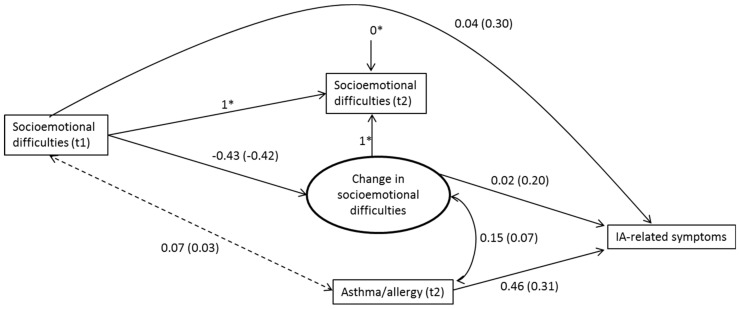
Unstandardized regression coefficients of latent change factor model: IA-related symptoms on psychosocial predictors and asthma/allergy variable (*N* = 986). Standardized values in parentheses. Dashed lines represent non-significant associations. * Fixed factors.

We started by analyzing two null models, one for each of our outcome variables [55]. We used a null model to estimate the variance between student and school levels and the intraclass correlation (ICC). The latter reports the proportion of the variance that belongs to the school level [55]. In order to test whether changes in psychosocial variables were associated with subjective IAQ and IA-related symptoms, we built several models for each outcome variable. First, we tested whether the psychosocial variables measured in seventh and ninth grades were associated with the outcome variables measured in ninth grade by using linear regression modelling in separate models. Second, we examined the degree to which the changes in psychosocial variables were associated with our outcome variables by using latent change modelling [52]. In a latent change model, the change is modelled directly through a latent difference factor, which means that in our models the psychosocial variables measured in ninth grade were perfectly explained by the predictors measured in seventh grade and the change factor. Except in Figure 1 and Figure 2, we report only standardized estimates (i.e., when the predictor increases by one standard deviation, the outcome variable increases by the standardized estimate [56]).

Gender and asthma/allergy variables had the lowest percentages of missing values (0%), and parents’ highest education had the highest (8%). We assumed that values were missing at random [57]. We dealt with the missing data by using a FIML estimation that produces unbiased values of parameters by determining the value that maximizes the likelihood function based on all available data [58].

## 3. Results

On average, students reported rather good subjective IAQ (Table 1). Furthermore, they reported IA-related symptoms less than once a month. There was a significant increase in students’ socioemotional difficulties (paired samples *t*-test: *t*(960) = 11.42, *p* < 0.001). Furthermore, the students reported more problems in teacher–student relations (paired samples *t*-test: *t*(948) = 4.47, *p* < 0.001) in ninth grade than in seventh grade. All the predictors were significantly correlated with subjective IAQ and IA-related symptoms (Table 2). Socioemotional difficulties measured in ninth grade had the highest correlations with the outcome variables. The intraclass correlations (ICCs) of the outcome variables were very low (subjective IAQ: ICC = 0.03; IA-related symptoms: ICC = 0.01). This means that the proportion of variance belonging to the school level was small. However, there were statistically significant variabilities between schools in both outcome variables (subjective IAQ: σ^2^_B_ = 0.02, *p* < 0.01; IA-related symptoms: σ^2^_B_ = 0.01, *p* < 0.05). Therefore, we used a sandwich estimator in the analysis to take into account the non-independence of observations (i.e., complex model in Mplus) [53].

### 3.1. Associations between Psychosocial Predictors and Subjective IAQ

We present four linear regression models in Table 3. In Model 1, we tested whether students’ socioemotional difficulties and perceived teacher–student relations measured in seventh grade related to their subjective IAQ measured in ninth grade. Even after the insertion of background variables, both psychosocial predictors measured in seventh grade were still significantly related to subjective IAQ measured in ninth grade. Socioemotional difficulties were the strongest predictor. There were no significant associations between background variables and subjective IAQ.

In Model 2, we tested whether students’ socioemotional difficulties and perceived teacher–student relations measured in ninth grade were associated with their subjective IAQ measured in the same grade. The standardized regression coefficients were higher than in Model 1, socioemotional difficulties still being the strongest predictor. There were no significant associations between background variables and subjective IAQ. Model 1 adjusted by background variables explained 6% of the total variance; Model 2 adjusted by background variables explained 12% of the total variance.

After all the main effects were analyzed, we tested whether the change in psychosocial predictors between seventh and ninth grades was related to subjective IAQ in ninth grade by using latent change modelling [52]. First, we included only the predictors—socioemotional difficulties and perceived teacher–student relations measured in seventh and ninth grades—in the same model. Socioemotional difficulties measured in seventh grade did not significantly predict the change in teacher–student relations between seventh and ninth grades. Conversely, perceived teacher–student relations measured in seventh grade did not significantly predict the change in socioemotional difficulties. The significant negative associations between the baseline levels and the change in our predictors indicated a higher increase in these predictors when the student’s baseline level was rather low, and conversely a lower increase when the student’s baseline level was rather high (socioemotional difficulties: standardized beta = −0.42, *p* < 0.001; teacher–student relations: standardized beta = −0.58, *p* < 0.001). This phenomenon is known as regression to the mean [59].

Next, we included our outcome variable, subjective IAQ in ninth grade, in the model (Figure 1). Since the background variables were not significantly associated with the outcome variable, we did not include them in the model. Changes in socioemotional difficulties and perceived teacher–student relations were positively and significantly related to subjective IAQ (socioemotional difficulties: standardized beta = 0.20, *p* < 0.001; teacher–student relations: standardized beta = 0.21, *p* < 0.001). This means that the more socioemotional difficulties students had in ninth relative to seventh grade, the more problems they reported in their school’s IAQ in ninth grade. Conversely, the more problems students reported in teacher–student relations in ninth relative to seventh grade, the more problems they reported in their school’s IAQ in ninth grade. The model explained 13% of the total variance.

### 3.2. Associations between Psychosocial Predictors and IA-Related Symptoms

In Model 3 we tested whether students’ psychosocial problems measured in seventh grade were associated with their IA-related symptoms measured in ninth grade (Table 4). In Model 4, we tested whether students’ psychosocial problems measured in ninth grade were associated with their IA-related symptoms measured in the same grade (Table 4). Psychosocial predictors and asthma/allergy were the only significant predictors in all models, except gender in Model 3. This association, however, was very weak, and it was not replicated in ninth grade. Asthma/allergy was the strongest predictor in both years. Model 3 adjusted by background variables explained 7% and Model 4 adjusted by background variables explained 19% of the total variance.

As above, we tested using latent change modelling whether the change in socioemotional difficulties and perceived teacher–student relations between seventh and ninth grades was related to IA-related symptoms in ninth grade. In the final model, we included only one psychosocial predictor in seventh and ninth grades—i.e., socioemotional difficulties—because perceived teacher–student relations were not significantly associated with IA-related symptoms in the earlier linear regression models (see Table 4). The change in socioemotional difficulties was positively and significantly related to IA-related symptoms (standardized beta = 0.23, *p* < 0.001): the more socioemotional difficulties students reported in ninth grade relative to seventh grade, the more IA-related symptoms they reported in ninth grade. The model explained 9% of the total variance. Next, given that asthma/allergy was a significant background variable, we included it in the model. We used only the asthma/allergy variable measured in ninth grade. The asthma/allergy variable measured in seventh grade was not a significant background variable once the variable measured in ninth grade was included. The change in socioemotional difficulties remained a significant predictor (standardized beta = 0.20, *p* < 0.001) (see Figure 2). The asthma/allergy variable measured in ninth grade was positively and significantly related to IA-related symptoms (standardized beta = 0.31, *p* < 0.001). The latter finding means that students who reported suffering from asthma, allergic rhinitis, or hay fever in ninth grade also reported significantly more IA-related symptoms in the same grade. There was a weak but significant correlation between the change in socioemotional difficulties and the asthma/allergy variable (partial correlation = 0.07, *p* < 0.05). This model explained 19% of the total variance.

## 4. Discussion

Our main aim was to analyze whether changes in students’ self-reported socioemotional difficulties and their perceptions of teacher–student relations were related to their subjective IAQ and IA-related symptoms. We found that the increase in socioemotional difficulties between seventh and ninth grades was significantly related to decreased subjective IAQ in ninth grade. Furthermore, we found that the increase in problems in perceived teacher–student relations between seventh and ninth grades was significantly related to decreased subjective IAQ in ninth grade. Finally, we found a significant relationship between increased socioemotional difficulties and IA-related symptoms in ninth grade.

To our knowledge, only one previous study has analyzed the associations between psychosocial problems and subjective IAQ among school-age children [32]. Our study supplements that cross-sectional study by showing that changes in psychosocial problems are related to subjective IAQ. Hence, it provides further support for the notion that psychosocial problems play a role in the evaluation of schools’ IAQ among students. Except for one cross-sectional study [33], previous IA research on the association between psychosocial problems and IA-related symptoms has focused on adults, and longitudinal studies are rare [5,30,60]. Our findings show that also schoolchildren’s psychosocial problems may influence the level of reported IA-related symptoms. Furthermore, our findings show that students’ histories of asthma and allergies play a role in this reporting. In total, our findings support the view that both physiological and psychosocial problems are associated with IA-related symptoms [37]. The literature suggests that stress related to psychosocial problems may induce IA-related symptoms [18,37,38]. However, the change in perceived teacher–student relations did not predict IA-related symptoms; nor were students’ histories of asthma and allergies related to subjective IAQ.

From the practical point of view, our results support the notion that when self-administered questionnaires are used to evaluate perceived IAQ among students in schools, items measuring psychosocial problems need to be included. Recent research shows that such questionnaires can be useful tools in assessing schools’ IAQ [6,61]. However, it is important to note that students’ psychosocial problems explained only 9–13% of the outcome variables’ total variances. This is in line with results provided by Finell and colleagues [32]. They found that four different psychosocial problems (e.g., teacher–student relations and school stress) and background variables explained 10% of subjective IAQ’s total variance on the student level. Bearing in mind that the evidence among the student population is very limited in this respect, these findings suggest that although psychosocial problems need to be taken into account, their role should not be exaggerated: most of the variance remains unexplained. Thus, a task for future research is to better map the other factors (such as unpleasant school environments, dirt, or poor lighting) that might influence students’ responses to IAQ questionnaires in contexts where there are no IA problems.

### Strengths, Limitations, and Future Research

The strengths of our study include the fact that we used longitudinal data; we used sophisticated statistical methods that took account of the non-independence of observations; and we measured socioemotional difficulties with a highly validated measure [62] that has not been used in previous IA research.

In this study, we focused only on schools with no known IA problems. Given that schools with IA problems may induce both health and psychosocial problems [8,39,63,64], it is possible that the relationship between students’ psychosocial problems, subjective IAQ and IA-related symptoms is more complex in such schools than in schools without IA problems. Therefore, it is important to analyze how these factors relate to each other in contexts where there are no building-related problems.

A limitation of the study is that we did not have physical measurements from the schools, which meant that we had to rely on school principals’ reports that inspectors had found no biological exposures in their schools. Furthermore, we did not know whether there were other kinds of problem in the schools’ indoor environments such as problems in ventilation, for example. However, IA-related symptoms were relatively rare among our participants. Furthermore, they reported rather good subjective IAQ. This provides further confirmation that we were indeed studying schools without IA problems. In the future, these issues should be further studied by using physical measurements and data which include at least three measurement points. Further research is also needed that uses experimental design and laboratory conditions in order to better understand these associations [65].

## 5. Conclusions

Our study showed that an increase in psychosocial problems—socioemotional difficulties and problems in perceived teacher–student relations—in school-age children was related to decreased subjective IAQ in the two-and-a-half-year follow-up. Furthermore, we showed that an increase in socioemotional difficulties was related to increased IA-related symptoms in the same period. This study supports the notion that psychosocial problems need to be taken into account when the quality of indoor environments is assessed by a questionnaire given to school students. A task for future research is to better understand the other factors that might influence how students answer IAQ questionnaires in schools without IA problems.

## Figures and Tables

**Table 1:** Descriptors of all used variables (*N* = 903–986).

	*N*	% Or Mean	SD ^2^	Min.	Max.
**Outcome variables ninth grade**					
Subjective IAQ ^1^	959	1.90	0.81	1	4
IA-related symptoms ^1^	971	1.66	0.63	1	4
**Predictors seventh grade**					
Teacher–student relations ^1^	973	2.60	0.76	1	5
Socioemotional difficulties ^1^	978	8.86	5.30	0	32
**Predictors ninth grade**					
Teacher–student relations ^1^	961	2.74	0.80	1	5
Socioemotional difficulties ^1^	968	10.88	5.74	0	30
**Background variables**					
Gender (female %)	495	50			
Ethnic background (Finnish- or Swedish-speaking %)	916	94			
Parents’ highest education (%)					
Low	52	6			
Middle	524	58			
High	327	36			
Asthma/allergy seventh grade (yes %)	132	13 ^3^			
Asthma/allergy ninth grade (yes %)	243	25 ^3^			

^1^ A higher value in a variable means more problems. ^2^ SD = standard deviation. ^3^ The difference between seventh and ninth grades was significant (*p* < 0.001). Note, the allergy was measured differently in seventh and ninth grades. Please see Section 2.3.3.

**Table 2:** Bivariate correlation coefficients of outcome variables (1–2) and psychosocial predictors (3–6).

	1	2	3	4	**5**
1. Subjective IAQ (t2)	1				
2. IA-related symptoms (t2)	0.26 ***	1			
3. Teacher–student relations (t1)	0.15 ***	0.09 *	1		
4. Socioemotional difficulties (t1)	0.22 ***	0.22 ***	0.30 ***	1	
5. Teacher–student relations (t2)	0.24 ***	0.10 *	0.26 ***	0.09 **	1
6. Socioemotional difficulties (t2)	0.30 ***	0.29 ***	0.16 ***	0.53 ***	0.19 ***

Correlations estimated using FIML with robust standard errors (*N* = 986). A higher value in a variable means more problems. *** *p* < 0.001, ** *p* < 0.01, * *p* < 0.05.

**Table 3:** Standardized regression coefficients and standard errors of linear regression Models 1 and 2 (*N* = 972–986). Outcome variable is subjective IAQ.

	Model 1: Predictors in Seventh Grade		Model 2: Predictors in Ninth Grade	
	1 ^f^	2 ^g^	1 ^f^	2 ^g^
	B (SE)	B (SE)	B (SE)	B (SE)
**Predictors seventh grade:**				
Teacher–student relations (t1) ^a^	0.10 (0.033) **	0.10 (0.033) **		
Socioemotional difficulties (t1) ^a^	0.19 (0.024) ***	0.18 (0.024) ***		
**Predictors ninth grade:**				
Teacher–student relations (t2) ^a^			0.19 (0.043) ***	0.19 (0.043) ***
Socioemotional difficulties (t2) ^a^			0.26 (0.032) ***	0.26 (0.034) ***
**Background variables:**				
Gender ^b^		−0.03 (0.030)		0.01 (0.028)
Ethnic background ^c^		−0.03 (0.026)		−0.02 (0.030)
Parents’ highest education ^d^		−0.03 (0.035)		−0.01 (0.036)
Asthma/allergy (t1) ^e^		0.04 (0.042)		
Asthma/allergy (t2) ^e^				0.04 (0.038)
R^2^	0.06	0.06	0.12	0.12

A higher value in the outcome variable means worse subjective IAQ. *** *p* < 0.001, ** *p* < 0.01, * *p* < 0.05. ^a^ A higher value in a variable means more problems. ^b^ Scale 0–1 (1 = boy). ^c^ Scale 0–1 (1 = language other than Finnish or Swedish). ^d^ Scale 0–2 (2 = high education). ^e^ Scale 0–1 (1 = asthma/allergy). ^f^ Model without background variables. ^g^ Model adjusted by background variables.

**Table 4:** Standardized regression coefficients and standard errors of linear regression Models 3 and 4 (*N* = 978–986). The outcome variable is IA-related symptoms.

	Model 3: Predictors in Seventh Grade		Model 4: Predictors in Ninth Grade	
	1 ^f^	2 ^g^	1 ^f^	2 ^g^
	B (SE)	B (SE)	B (SE)	B (SE)
**Predictors seventh grade:**				
Teacher–student relations (t1) ^a^	0.02 (0.042)	0.03 (0.044)		
Socioemotional difficulties (t1) ^a^	0.22 (0.031) ***	0.21 (0.030) ***		
**Predictors ninth grade:**				
Teacher–student relations (t2) ^a^			0.04 (0.037)	0.05 (0.036)
Socioemotional difficulties (t2) ^a^			0.29 (0.037) ***	0.26 (0.038) ***
**Background variables:**				
Gender ^b^		−0.06 (0.026) *		−0.04 (0.030)
Ethnic background ^c^		0.01 (0.042)		0.02 (0.034)
Parents’ highest education ^d^		0.03 (0.036)		0.06 (0.033)
Asthma/allergy (t1) ^e^		0.12 (0.042) **		
Asthma/allergy (t2) ^e^				0.32 (0.038) ***
R^2^	0.05	0.07	0.09	0.19

A higher value in the outcome variable means more IA-related symptoms. *** *p* < 0.001, ** *p* < 0.01, * *p* < 0.05. ^a^ A higher value in a variable means more problems. ^b^ Scale 0–1 (1 = boy). ^c^ Scale 0–1 (1 = language other than Finnish or Swedish). ^d^ Scale 0–2 (2 = high education). ^e^ Scale 0–1 (1 = asthma/allergy). ^f^ Model without background variables. ^g^ Model adjusted by background variables.
